# Spatial Distribution Characteristics and Human Health Risk Assessment of Organophosphate Esters in Indoor Dust in Beijing

**DOI:** 10.3390/toxics14070629

**Published:** 2026-07-20

**Authors:** Huizi Yuan, Ziyan Chen, Weicheng Zhao, Mingyang Li, Yinghong Wang, Xingru Li, Fangkun Wu

**Affiliations:** 1 College of Resource Environment and Tourism, Capital Normal University, Beijing 100048, China; m17399116443@163.com (H.Y.); 16645716744@163.com (M.L.); 2 Analysis and Testing Center, Department of Chemistry, Capital Normal University, Beijing 100048, China; zy.chen27@foxmail.com (Z.C.); 1200703021@cnu.edu.cn (W.Z.); 3 Institute of Atmospheric Physics, Chinese Academy of Sciences, Beijing 100029, China; wangyinghong@mail.iap.ac.cn

**Keywords:** indoor dust, organophosphate esters, health risk assessment, PMF source apportionment, correlation analysis

## Abstract

Organophosphate esters (OPEs), extensively employed as flame retardants and plasticizers, have emerged as ubiquitous contaminants in indoor environments following the phase-out of brominated flame retardants. However, the occurrence patterns, source characteristics, and exposure implications of emerging OPE congeners across diverse urban indoor microenvironments remain inadequately elucidated. In this study, twenty-eight OPE congeners were quantified in settled dust collected from eight representative indoor microenvironments and one outdoor reference site in Beijing, China. Total OPE concentrations exhibited pronounced spatial heterogeneity, ranging from 8.46 to 42.60 mg kg^−1^, with the highest levels observed in subway stations and laboratories. Non-halogenated OPEs dominated the contamination profile, accounting for 80.7% of the total OPEs, whereas Tris(2-ethylhexyl) phosphate (TEHP), 2-Ethylhexyl diphenyl phosphate (EHDPP), Tris(1-chloro-2-propyl) phosphate (TCIPP), Tris(2-chloroethyl) phosphate (TCEP), and Triphenyl phosphate (TPhP) collectively contributed 85.1% of the overall burden. Correlation analysis and positive matrix factorization revealed that indoor OPE contamination was primarily associated with emissions from building materials, polymer-containing consumer products, furniture, and electronic equipment. Transformation-associated compounds such as Bis(2-butoxyethyl) hydroxyethyl phosphate (BBOEHEP) were detected in indoor dust, pointing to the plausible presence of OPE transformation processes in such enclosed settings. Exposure assessment demonstrated that dust ingestion accounted for over 98% of total OPE intake, resulting in substantially higher exposure levels in children than in adults. Although estimated non-carcinogenic and carcinogenic risks remained below established health-based thresholds, exposure was disproportionately driven by a limited number of congeners, particularly EHDPP, TCIPP, TCEP, and TEHP. These findings highlight the growing contribution of replacement OPEs to indoor chemical burdens and underscore the importance of incorporating transformation products and indoor aging processes into future exposure and risk assessments frameworks.

## 1. Introduction

Organophosphate esters (OPEs) have become one of the most widely used classes of flame retardants and plasticizers following the global phase-out of brominated flame retardants (BFRs) [1,2]. Unlike reactive additives that are chemically bonded to polymer matrices, OPEs are primarily incorporated into consumer products through physical blending. Consequently, they can continuously migrate from materials into surrounding environments via volatilization, abrasion, and surface leaching. As a result, OPEs have been ubiquitously detected in indoor air, settled dust, water, and biota, raising increasing concerns regarding chronic human exposure [3,4]. Indoor environments are of particular importance because modern populations spend more than 80% of their time indoors, where building materials, furniture, textiles, and electronic products collectively constitute substantial reservoirs and emission sources of OPEs.

Growing evidence indicates that OPE exposure may induce a range of adverse biological effects, including endocrine disruption, neurotoxicity, reproductive toxicity, developmental abnormalities, and potential carcinogenicity [5,6,7]. Biomonitoring studies have reported the widespread occurrence of OPEs and their metabolites in human serum, urine, breast milk, hair, and blood, demonstrating extensive internal exposure among the general population [8,9,10,11,12,13]. Among multiple exposure pathways, indoor dust has been recognized as a major carrier of OPEs because these semi-volatile compounds preferentially partition into particulate matter and accumulate in indoor reservoirs. A study confirmed that long-term exposure to OPEs can induce endocrine disruption, liver and kidney damage, neurological dysfunction, and even potential carcinogenic risks [14]. Targeted toxicity studies on typical monomers further indicate that triphenyl phosphate (TPhP) has been shown to increase blood–brain barrier permeability, induce neuroinflammatory responses, and downregulate mRNA expression levels in fish brain tissue; simultaneously, aryl organophosphates represented by TPhP, 2-ethylhexyl diphenyl phosphate (EHDPP), and trichlorophosphate (TCrP) induce reproductive toxic effects by disrupting hormonal balance and interacting with nuclear receptors, particularly the estrogen receptor α [15].

Surveys on OPEs in indoor dust have several unaddressed research limitations. Most monitoring campaigns target only a narrow range of parent OPE monomers, with few simultaneously detecting diverse homologues and environmental transformation products. Such limited analyte coverage may underestimate total OPE mixture loads and latent health risks indoors. In sampling layout, most published investigations center on one single indoor microenvironment like homes or offices, hindering full clarification of city-scale spatial variation in OPE occurrence. For instance, Cai et al. [16] quantified merely 10 OPE congeners across four indoor functional zones in Guangzhou, while Solanke et al. [17] measured 13 target OPEs in indoor matrices across Tampa Bay. Dong et al. [18] focused exclusively on OPE concentrations, sources and contributions within air and dust from new vehicle cabins. In health risk appraisal, many assessments adopt default U.S. EPA exposure parameters, which show mismatches with the daily activity patterns and physiological baselines of Chinese residents. Studies exploring age- and gender-stratified exposure disparities remain scarce. Related works include a neonatal risk assessment by Foster et al. [19], as well as research by Persson et al. [20] addressing child exposure to brominated flame retardants during preschool attendance.

To address these gaps, this study quantified 28 OPEs in settled dust collected from eight representative indoor microenvironments and an outdoor reference site in Beijing, China. The specific objectives were to: (i) characterize the occurrence patterns and spatial distribution of OPEs across different indoor environments; (ii) identify potential emission sources and quantify their contributions using correlation analysis and positive matrix factorization (PMF); and (iii) evaluate age- and gender-specific exposure and health risks using exposure parameters derived from Chinese population datasets. This work provides an integrated assessment linking indoor contamination, source characteristics, and human exposure, thereby improving the understanding of OPE behavior and associated health implications in urban indoor environments.

## 2. Materials and Methods

### 2.1. Sample Collection

Sampling sites were centered on Capital Normal University and radiated outward to surrounding typical urban functional spaces. The study area falls within Haidian District, the core urban zone of Beijing, which features dense population, diversified land-use patterns, and intensive anthropogenic activities.

Dust samples were collected from eight indoor microenvironments in Beijing, including residences, dormitories, classrooms, libraries, laboratories, stairwells, restaurants, and subway stations, together with one outdoor reference site. These sampled microenvironments cover distinct activity domains of research, teaching, residence, public services and transportation, sufficiently capturing the multi-medium, multi-scenario exposure patterns of urban residents throughout daily occupational and domestic activities. Sampling was conducted using a Xiaomi portable vacuum sampler fitted with a high-efficiency particulate air (HEPA) filtration unit (manufactured by Ningbo Fujia Industrial Co., Ltd., Ningbo, Zhejiang, China) to ensure efficient particle capture and minimize cross-contamination. A multi-point composite strategy was applied at each site, with at least five subsamples obtained from representative surfaces and combined to form a single integrated sample.

To reduce potential contamination during collection, all procedures were performed using nitrile gloves, and contact with plastic materials was minimized throughout the sampling process. Samples were wrapped in pre-cleaned aluminum foil immediately after collection, transported to the laboratory under cooled conditions, and stored at 4 °C prior to extraction and instrumental analysis. In total, nine typical microenvironment categories were covered (*n* = 3 samples per category), with 27 sampling sites established across all locations, yielding a total of 135 dust samples (approximately 10% field duplicates). Field duplicates were deployed to assess sampling reproducibility and analytical consistency.

### 2.2. Sample Preparation and Analysis

Prior to extraction, dust samples were sieved to isolate the fine particle fraction (<45 μm), which is considered the most relevant size range for assessing human exposure via ingestion and dermal contact pathways. Approximately 100 mg of each homogenized sample was subjected to methanol-assisted ultrasonic extraction. The extracts were subsequently centrifuged and filtered to remove particulate residues, followed by solvent reduction under a gentle nitrogen stream prior to instrumental analysis.

Qualitative and quantitative analysis of sample extracts was performed using an ultra-performance liquid chromatography-tandem mass spectrometry (UPLC–MS/MS) system consisting of an ACQUITY UPLC (Waters, Milford, MA, USA) and a 6500 QTRAP triple quadrupole linear ion trap mass spectrometer (SCIEX, Framingham, MA, USA). Twenty-eight target organophosphate ester (OPE) standards were included in the analytical suite. External calibration was applied for quantification. High-purity LC-grade methanol, acetonitrile, and formic acid were used throughout all preparation and analytical procedures to ensure analytical consistency and minimize background contamination. Chromatographic separation conditions, mass spectrometric parameters, target compound information, and standard details are provided in Supplementary Text S1 and Table S1.

### 2.3. Quality Control and Assurance

Calibration curves were constructed at eight concentration levels, and all target compounds exhibited strong linear responses across the tested ranges (*R*^2^ > 0.99), indicating robust instrumental performance and stable quantification behavior. Method detection limits (MDLs) varied from 0.027 to 0.960 μg mL^−1^, reflecting compound-dependent sensitivity differences associated with ionization efficiency and instrumental response. Analytical accuracy and precision were evaluated using recovery experiments and replicate measurements. Mean recovery values ranged from 80.0% to 119.3%, while relative standard deviations (RSDs) remained below 10.2%, demonstrating acceptable method reproducibility across target analytes with diverse physicochemical properties.

Quality assurance and quality control (QA/QC) procedures included field blanks, procedural blanks, and duplicate samples, which were systematically incorporated throughout the analytical workflow to monitor potential contamination and analytical variability. A detailed summary of QA/QC performance metrics is provided in Tables S2 and S3.

### 2.4. Source Apportionment

Potential sources of OPEs were identified using Positive Matrix Factorization (PMF, EPA PMF 5.0). Concentration data and associated uncertainty matrices were used as model inputs. Multiple factor solutions were evaluated based on Q values, residual distributions, and interpretability of source profiles. The optimal factor solution was selected to characterize source contributions and spatial variations among indoor environments. Detailed uncertainty calculations and model settings are provided in Supplementary Text S2.

### 2.5. Health Risk Assessment

Human exposure to organophosphate esters (OPEs) associated with indoor dust was estimated by considering three exposure pathways, including dust ingestion, inhalation, and dermal contact. Average daily doses (ADDs) were calculated for four age groups, namely children (Todd., 3–6 years), adolescents (Adol., 7–19 years), adults (Adu., 20–59 years), and elderly individuals (Eld., 60–79 years), to capture age-dependent variability in exposure behavior and physiological susceptibility.

Compared with previous assessments that primarily relied on default exposure factors derived from U.S. EPA guidelines, this study applied population-specific parameters from the Chinese Exposure Factors Handbook and relevant national risk assessment frameworks. This adjustment reduces potential bias associated with cross-population extrapolation and provides exposure estimates that are more representative of behavioral patterns, body weight distributions, and activity characteristics of the Chinese population. However, uncertainties remain due to variability in individual behavior and limited availability of compound-specific bioaccessibility data.

Non-carcinogenic risk was evaluated using hazard quotients (HQs) and hazard indices (HIs), while carcinogenic risk (CR) was estimated for compounds with established cancer slope factors. This risk characterization framework provides a screening-level assessment of potential health concern associated with chronic exposure to OPEs in indoor environments. Detailed computational formulations, exposure parameters, and toxicological reference values are provided in Supplementary Texts S3 and S4 and Tables S4 and S5 [21,22,23,24,25,26,27,28,29,30,31,32,33].

### 2.6. Statistical Analysis

Concentrations below MDL were replaced with half of the corresponding limit, and raw OPE concentrations were log_10_-transformed to reduce skewness. All statistical calculations and plotting were conducted using Origin Pro 2022. Results of each sampling site were expressed as mean ± standard deviation (SD) with 95% two-sided confidence intervals (95% CI). Shapiro–Wilk normality test and Levene’s homogeneity test were performed before parametric analysis. One-way ANOVA coupled with Tukey’s HSD post hoc multiple comparison was used to compare log_10_(OPE) levels across nine indoor microenvironments, and *p* < 0.05 was defined as the threshold of statistical significance. All sampling sites shared the same homogeneous group letter “A”, demonstrating no significant pairwise inter-site differences. full descriptive statistics are summarized in Tables S6–S8 and Figure S1 of Supplementary Materials.

Multiple diagnostic metrics were adopted to validate PMF model reliability, including Q-statistics of convergent model runs, scaled residual distribution, and bootstrap factor mapping results, with all relevant data summarized in Tables S9 and S10 and Figure S1 in the Supplementary Materials.

## 3. Results and Discussion

### 3.1. Concentration and Distribution Characteristics of OPEs

**Figure 1:**
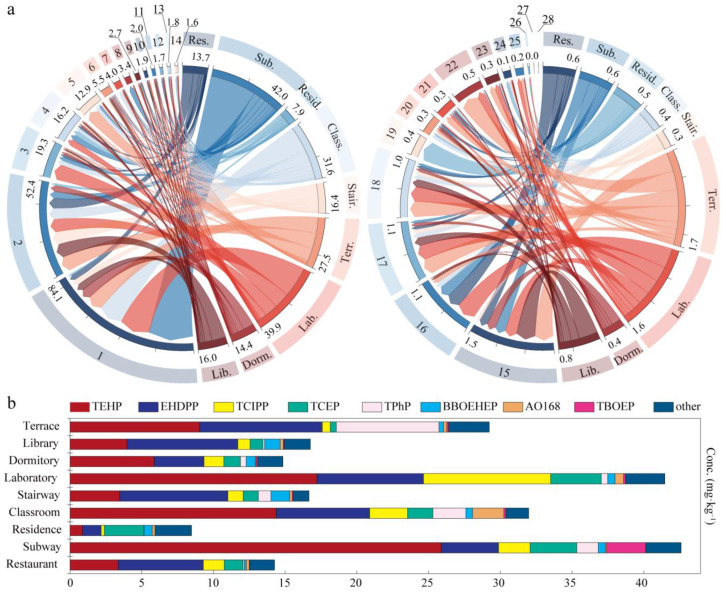
Composition and distribution characteristics of OPEs in different indoor environments. (**a**) Concentrations of 28 OPEs and their distribution across sampled indoor microenvironments. 1-TEHP; 2-EHDPP; 3-TCIPP; 4-TCEP; 5-TPhP; 6-BBOEHEP; 7-AO168; 8-TBOEP; 9-AO168=O; 10-DBzP; 11-DMTP.Na; 12-DMP; 13-TDCIPP; 14-TEP; 15-4-OH-BDE-47; 16-TMPP; 17-TiBP; 18-TnBP; 19-AO626; 20-MDPP; 21-TiPP; 22-TnPP; 23-V6; 24-TnPPi; 25-TDBPP; 26-TMP; 27-3-OH-TnBP; 28-TPHPi. (**b**) Concentration profiles of the 28 OPEs in 9 indoor environments.

Twenty-eight organophosphate esters (OPEs) were quantified in indoor dust collected from multiple urban microenvironments in Beijing, as illustrated in Figure 1a,b and Table S11. Total concentrations varied between 8.46 and 42.60 mg kg^−1^, indicating non-uniform accumulation across functional indoor spaces. Elevated levels were observed in subway stations, laboratories, and classrooms, suggesting that indoor OPE burdens are influenced by the composition of material inventories and the intensity of polymer use within specific environments, rather than by human occupancy alone.

When compared with previously reported data from North America and Western Europe, the concentration levels observed in this study fall within the lower range of highly industrialized regions but exceed those reported in several developing countries, including Nepal and India [34,35,36,37]. However, such cross-regional comparisons should be interpreted with caution due to differences in target compound lists, analytical coverage, and sampling strategies. Therefore, observed variations are more likely to reflect differences in product usage patterns, building material composition, and regulatory frameworks governing flame retardants, rather than differences in regional atmospheric background levels.

Spatial differences among microenvironments further support the dominance of indoor source structures. Concentrations varied by approximately fivefold across sampling sites, from 8.46 mg kg^−1^ in residential environments to 42.60 mg kg^−1^ in subway stations, with similarly elevated levels in laboratories (41.46 mg kg^−1^) and classrooms (31.97 mg kg^−1^). These patterns indicate that OPE accumulation is controlled primarily by source density and material-specific emissions, particularly from electronic devices, polymer-based construction materials, synthetic furnishings, and cable insulation systems.

Rather than representing short-term contamination events, these distributions are consistent with a time-integrated accumulation process in which indoor dust functions as a reservoir for semi-volatile additives continuously released from material stocks. However, this reservoir interpretation does not imply equilibrium conditions, as ongoing emissions, ventilation variability, and surface–air partitioning collectively maintain a dynamic and non-steady-state system.

The contamination profile was strongly skewed toward a limited number of compounds. TEHP, EHDPP, TCIPP, TCEP, and TPhP collectively contributed 85.05% of total OPE concentrations, indicating that indoor exposure is governed by a small subset of dominant congeners despite the large number of commercially available phosphate esters. This uneven distribution suggests that exposure potential is not evenly distributed across compound classes, but is instead controlled by a limited group of chemicals with favorable emission and partitioning characteristics.

Compared with earlier studies conducted during the initial phase of OPE substitution, when chlorinated compounds such as TCEP and TDCIPP were dominant [38,39,40], the present composition reflects a clear shift in dominant chemical classes. Alkyl- and aryl-substituted OPEs accounted for 48.94% and 31.76% of total concentrations, respectively, whereas halogenated congeners represented only a minor fraction. This compositional transition is consistent with changes in global flame-retardant usage patterns following regulatory restrictions on several chlorinated formulations. However, whether this shift is driven primarily by changes in emission intensity or by differences in environmental persistence cannot be fully resolved based on concentration data alone. Recent monitoring studies have similarly reported increasing prevalence of aryl- and high-molecular-weight alkyl phosphate esters in indoor dust, supporting a broader transition toward replacement OPE formulations [17,41].

Among individual compounds, TEHP and EHDPP were the most abundant, with concentrations of 9.38 and 5.49 mg kg^−1^, respectively, followed by TCIPP, TCEP, and TPhP. The prominence of TEHP and EHDPP is notable because both compounds are increasingly recognized as replacement flame retardants with distinct physicochemical properties relative to legacy chlorinated OPEs. Their relatively high hydrophobicity and low vapor pressure favor partitioning into the particle phase and subsequent retention in settled dust, which may enhance their persistence in indoor reservoirs. As a result, these compounds may contribute disproportionately to long-term exposure even in the absence of dominant emission sources.

Comparative surveys across global indoor environments reveal region-specific OPE compositional signatures, which are largely shaped by differentiated furniture flammability codes and additive substitution pathways after global PBDE phase-out. Halogenated OPEs (TCIPP, TDCIPP) dominate indoor dust in Western Europe (Barcelona [42], Stockholm [43]) and North America, driven by mandatory flame retardation standards for upholstered polyurethane foams. Australia’s flooring industry relies heavily on TBOEP as brominated flame retardant substitutes, leading to TBOEP-predominant dust profiles [44], while mixed OPE mixtures (TBOEP, TPHP, TCIPP, TDCIPP) prevail in Brazil due to coexistence of imported flame-retarded furniture and local decorative materials [45]. Weak chemical supervision and low-cost thermal insulation additives make TCEP the dominant OPE in South African households [46]. Distinct from above global patterns, alkyl TEHP and aryl EHDPP jointly account for 63.57% of total OPEs in Beijing indoor dust, with TCIPP only contributing 9.98%. Such a unique fingerprint aligns with the source apportionment results in this work: construction and decorative materials serve as the dominant OPE reservoir, whereas chlorinated flame-retarded foams contribute limited pollution loads. This regional divergence can be partially explained by China’s fire safety regulatory framework, where flammability requirements are only enforced for public-space furniture rather than residential soft furnishings. Without mandatory halogenated OPE addition for domestic furniture, local decoration markets prioritize non-halogen alkyl/aryl OPE plasticizers for paints, sealants and flooring materials. Meanwhile, long-term window closure during Beijing’s centralized heating period reduces air exchange, and low volatility of TEHP and EHDPP facilitates their continuous accumulation in settled dust. From a regulatory perspective, the uneven shift toward alternative OPEs highlights a universal governance gap: chemical restriction policies commonly lag behind the market application of novel replacement additives. Current domestic standards fail to set limit thresholds for TEHP and EHDPP, which have become major indoor exposure contributors, suggesting targeted restrictions on plasticizer additives in decorative materials are necessary to mitigate residential OPE exposure risks.

The occurrence of EHDPP warrants particular attention. Although historically less regulated than chlorinated OPEs such as TCEP and TDCIPP, EHDPP has been increasingly reported as a major component of indoor dust in recent surveys [47]. Its widespread use in electronic devices, polymer coatings, and engineering plastics suggests multiple potential emission pathways across different indoor materials. Emerging toxicological evidence further indicates that EHDPP may exhibit endocrine-disrupting and developmental effects comparable to those of several regulated OPEs [47]. Taken together, the elevated abundance of EHDPP observed in this study suggests a gradual shift in indoor chemical burden from legacy chlorinated OPEs toward replacement phosphate esters whose environmental behavior and long-term health implications remain insufficiently characterized.

Collectively, these findings indicate that contemporary indoor OPE contamination can no longer be sufficiently explained by a framework centered on chlorinated flame retardants. The increasing dominance of TEHP and EHDPP suggests a progressive restructuring of indoor chemical profiles, driven by the ongoing transition toward replacement phosphate ester formulations. This shift implies that current monitoring and risk assessment approaches based primarily on legacy compounds may not fully capture the evolving composition of indoor OPE mixtures, underscoring the importance of incorporating emerging replacement chemicals into future analytical and exposure frameworks.

### 3.2. Source-Specific Chemical Signatures and Indoor Accumulation Mechanisms

**Figure 2:**
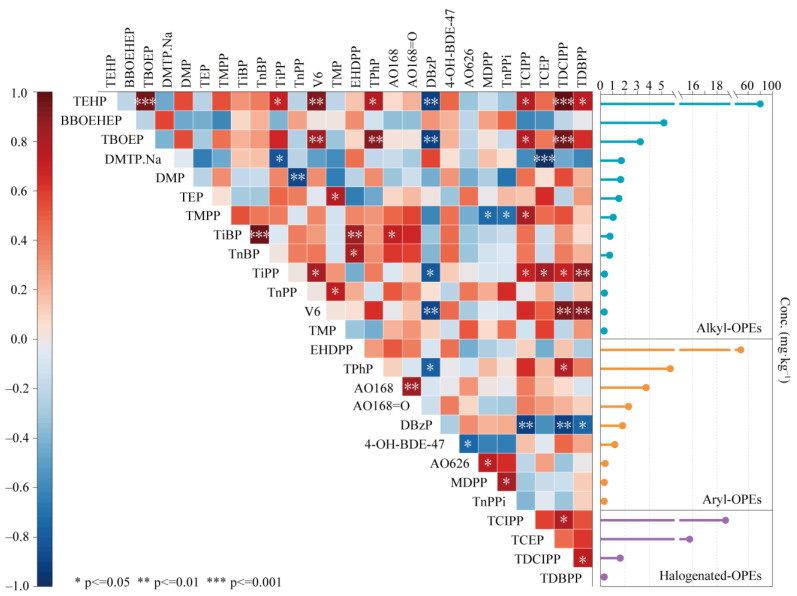
Correlation heatmap and concentration distribution of OPEs and co-occurring pollutants. The heatmap shows Spearman correlation coefficients, with asterisks indicating significance levels (*p* ≤ 0.05, *p* ≤ 0.01, *p* ≤ 0.001). The right panel displays concentrations of different classes of OPEs (Alkyl, Aryl, and Halogenated OPEs).

To further examine the factors governing the distribution of OPEs in indoor dust, Spearman correlation analysis was conducted across the 28 target compounds, as shown in Figure 2. The resulting correlation structure does not support a single dominant emission pathway, but instead reflects overlapping contributions from multiple source categories and environmental processes that jointly shape indoor OPE mixtures. Chlorinated OPEs, including TCIPP, TCEP, and TDCIPP, exhibited strong positive inter-correlations, suggesting shared associations with flame-retarded construction materials, polyurethane foams, and insulation systems. Similar clustering patterns have been widely reported in indoor environments and are generally attributed to co-formulation practices in building products designed to meet fire-safety regulations [3]. However, their relatively limited contribution to total OPE burden in this study suggests that chlorinated compounds may no longer represent the primary drivers of current indoor contamination profiles.

In contrast, aryl-substituted OPEs formed a distinct correlation module. EHDPP and TPhP showed particularly strong associations, consistent with their use in electronic devices, coatings, cable insulation, and engineering plastics. This pattern aligns with recent observations indicating an increasing contribution of aryl phosphate esters to indoor contamination following the phase-out of certain brominated flame retardants [48]. The higher abundance of EHDPP relative to TPhP further suggests that replacement formulations may be reshaping indoor chemical compositions rather than simply substituting legacy compounds on a one-to-one basis.

Beyond class-specific clustering, the correlation network highlights a broader mixture-driven structure. The largest interconnected cluster, centered on TBOEP, TDCIPP, TEHP, DBzP, and TiPP, indicates extensive co-occurrence among compounds associated with polymer-rich materials. Such patterns are more consistent with simultaneous release from multi-additive formulations embedded in consumer and building materials than with emission from isolated sources. However, correlation patterns alone cannot distinguish between co-formulation, co-location of materials, or shared environmental partitioning behavior, which introduces uncertainty in direct source attribution.

**Figure 3:**
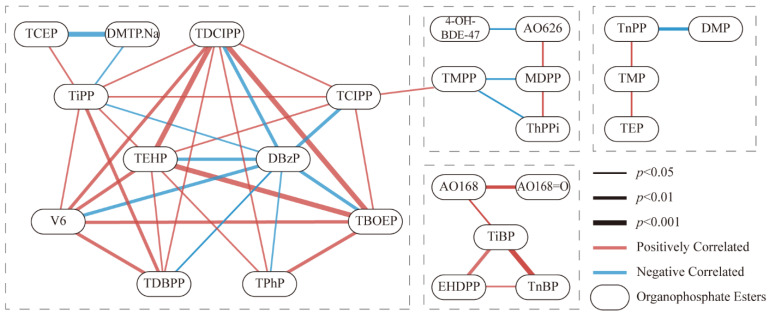
Spearman correlation network of OPEs and additive-derived compounds detected in indoor dust. Red and blue lines indicate significant positive and negative correlations, respectively. Line thickness represents correlation significance. Distinct clusters reflect shared emission sources, co-occurrence patterns, and potential transformation relationships among indoor contaminants (*p* < 0.05, *p* < 0.01, and *p* < 0.001).

Several low-abundance compounds exhibited relatively high network connectivity despite contributing minimally to total mass concentrations (Figure 3). This decoupling between concentration magnitude and network centrality suggests that compound behavior in indoor dust is not solely governed by abundance, but is also influenced by factors such as environmental persistence, partitioning behavior, and potential transformation processes. Accordingly, source interpretation based exclusively on dominant compounds may overlook structurally informative but low-concentration congeners that contribute disproportionately to the overall connectivity of the chemical network. However, correlation-based connectivity does not establish mechanistic linkage, and therefore should not be interpreted as direct evidence of shared emission sources.

Within the same network, significant positive associations were observed among aryl-substituted OPEs, particularly TPhP and EHDPP. These compounds are commonly associated with electronic devices, polymer coatings, engineering plastics, and synthetic material applications, suggesting that their co-occurrence may arise from their widespread use in similar categories of indoor materials. While this pattern is consistent with emissions from electronic and polymer-rich environments, attribution remains inferential, as co-occurrence may also be influenced by overlapping product usage and shared partitioning behavior in indoor environments. Nevertheless, the observed clustering provides support for the notion that OPE composition contains source-relevant structural information, although its interpretability is constrained by the non-specific nature of correlation-based analyses.

### 3.3. PMF-Derived Sources and Indoor Reservoir Effect

**Figure 4:**
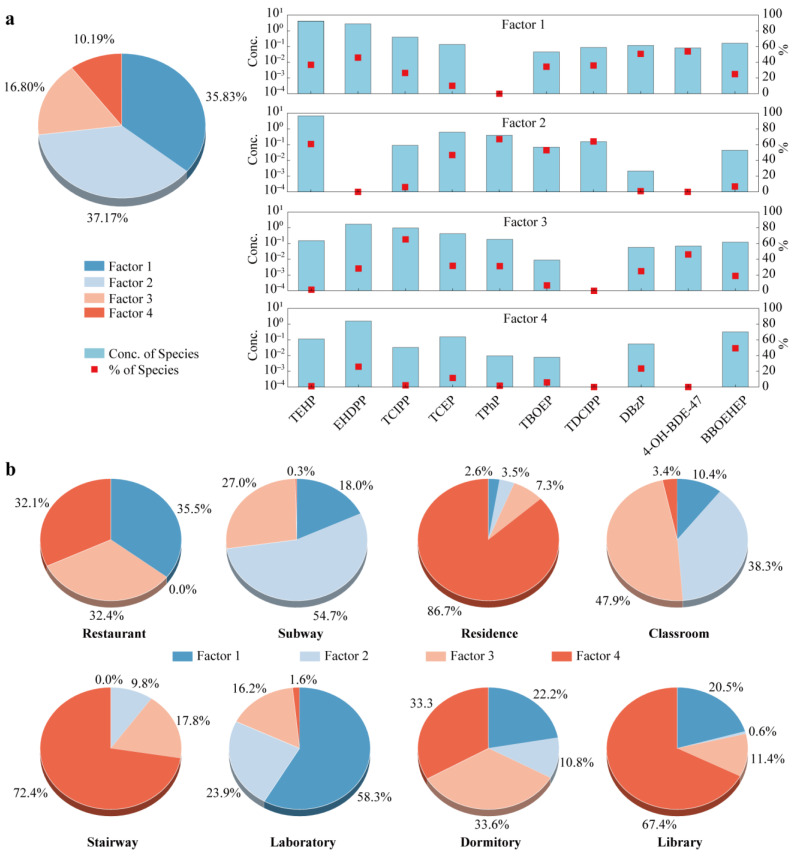
Source apportionment of OPEs using PMF model. (**a**) Factor profiles and contributions of each OPE congener to the four identified factors; (**b**) Spatial variations in the contributions of the four factors across different microenvironments. Note: Percentages are rounded to one decimal place, and minor deviations from 100% are inevitable as a result of rounding.

Positive matrix factorization (PMF) was applied to quantitatively resolve the major contributors to OPE contamination in indoor dust (Figure 4). A four-factor solution was selected based on statistical performance and interpretability of resolved profiles, explaining most of the variance in measured concentrations. The resulting factors represent statistically distinct compositional patterns; however, their interpretation as discrete emission sources should be considered with caution given the inherently mixed and co-located nature of indoor material emissions.

In contrast to outdoor atmospheric environments, where contaminant variability is often influenced by regional transport and episodic emission events, indoor OPE profiles are primarily shaped by continuous emissions from polymer-rich material stocks distributed across confined microenvironments [49]. Under such conditions, PMF-derived factors are more appropriately interpreted as integrated signatures reflecting combined effects of source emissions, material aging, and phase partitioning processes rather than strictly separated source categories. Therefore, the resolved factors likely capture both emission characteristics and longer-term indoor reservoir dynamics, in which semi-volatile additives accumulate and redistribute among air, surfaces, and settled dust over extended time scales. This dual interpretation highlights the limitation of applying conventional source apportionment frameworks developed for outdoor aerosols directly to indoor environments, where emission and accumulation processes are inherently coupled [38].

Factor 1 accounted for 35.83% of the total variance and was characterized by elevated loadings of DBzP and 4-OH-BDE-47. The highest contributions of this factor were observed in laboratories and restaurants, where relative contributions reached 58.3% and 35.5%, respectively. Rather than representing emissions from a single product category, this factor is more consistently interpreted as reflecting emissions associated with polymer-rich consumable materials. Such materials are subject to frequent handling, abrasion, and replacement, which collectively generate a continuous input of additive-derived compounds into indoor dust [3]. This mechanism suggests that short-lived or frequently replaced plastic products may still function as persistent emission vectors due to their high turnover rate. In this context, indoor contamination differs fundamentally from outdoor systems, where emissions are often governed by discrete point sources or episodic release events, whereas indoor environments are sustained by continuous material turnover and use cycles [50].

Factor 2 represented the largest contribution (37.17%) and exhibited a chemical profile dominated by compounds typically associated with construction and decorative materials [3,51]. This factor showed consistently high contributions across nearly all microenvironments, indicating that building-related sources constitute a pervasive baseline of indoor OPE contamination. Modern indoor spaces contain large inventories of polymer-modified paints, adhesives, sealants, insulation materials, decorative laminates, and synthetic flooring systems. Owing to their long service lifetimes, these materials act as semi-permanent reservoirs that continuously release OPEs through slow volatilization, diffusion, surface wear, and material aging processes [51]. However, the reservoir interpretation should not be understood as equilibrium storage; instead, it reflects a dynamic balance between ongoing emissions and limited removal processes under confined indoor conditions. The widespread presence of this factor suggests that building materials provide a persistent background source of exposure that is largely independent of short-term occupant activity.

Factor 3 explained 16.80% of the total variance and was primarily associated with TCIPP and TCEP. The highest contributions were observed in classrooms and dormitories, which contain substantial amounts of polyurethane foam-based furniture, mattresses, and upholstered materials. The chemical profile of this factor is consistent with historical application of chlorinated OPEs in flexible polyurethane foam products. However, despite this clear compositional signature, the contribution of chlorinated OPEs to the total OPE burden was substantially lower than that of alkyl- and aryl-substituted compounds. This pattern is consistent with an ongoing transition in indoor chemical composition, where legacy chlorinated flame retardants are progressively replaced by alternative formulations in response to regulatory and market pressures [17]. Nevertheless, the persistence of TCIPP and TCEP indicates that legacy materials remain an important, albeit declining, component of indoor contamination.

Factor 4 contributed 10.19% of the total variance and was enriched in EHDPP, TPhP, AO168 derivatives, and other aryl phosphate esters. Elevated contributions of this factor were observed in subway stations and laboratories, environments characterized by high densities of electronic devices, electrical infrastructure, and engineered polymer systems. The composition of this factor is particularly relevant because it reflects the growing influence of replacement organophosphate esters in indoor environments. EHDPP has recently been identified as one of the dominant phosphate esters in indoor dust across multiple studies, yet its sources and environmental behavior remain comparatively under-characterized relative to traditional chlorinated flame retardants. The association of EHDPP with electronics-rich environments suggests that the increasing penetration of electronic technologies and advanced polymer materials may be contributing to the accumulation of replacement OPEs within indoor reservoirs.

Beyond source attribution, the PMF results also provide insight into post-emission environmental behavior. Indoor environments differ fundamentally from outdoor atmospheric systems in that reduced ventilation, limited photochemical degradation, and continuous emissions collectively favor the accumulation of semi-volatile organic compounds. Following emission, OPEs partition among air, surfaces, and settled dust according to compound-specific physicochemical properties. High-molecular-weight compounds such as TEHP and EHDPP preferentially partition into particle-associated phases due to their low volatility and higher hydrophobicity, facilitating long-term retention in dust reservoirs. Consequently, indoor dust should be interpreted as a time-integrated environmental compartment rather than a passive receptor.

The pronounced enrichment of replacement OPEs within dust reservoirs further suggests that exposure potential is not solely determined by emission strength, but also by environmental persistence and partitioning behavior. Compounds such as EHDPP may therefore contribute disproportionately to long-term exposure despite the absence of identifiable point sources, as their physicochemical properties favor accumulation under confined indoor conditions. This reservoir-driven accumulation mechanism provides a plausible explanation for the consistently high abundance of EHDPP across multiple microenvironments and highlights the importance of incorporating environmental retention processes into future exposure and risk assessment frameworks.

Nevertheless, although PMF modeling and correlation analysis can preliminarily identify potential OPE emission sources, their inherent drawbacks including subjective factor selection, neglect of dynamic indoor chemical processes, and difficulty in separating independent contributions from coexisting material sources may introduce biases to the source apportionment results.

### 3.4. Evidence for Indoor Transformation Chemistry and BBOEHEP Formation

While primary emissions remain the dominant source of indoor organophosphate ester (OPE) contamination, some laboratory simulation results hypothesize that slow secondary chemical reactions may alter the composition, persistence and toxic potential of indoor OPE mixtures under long-term aging conditions. Traditionally, indoor dust has been conceptualized as a passive sink for contaminants emitted from consumer products and building materials. However, several laboratory simulations propose that indoor interfaces may create mild reaction conditions, where oxidation, hydrolysis and material aging could potentially shift pollutant composition over time [52,53].

Within this context, the detection of BBOEHEP represents a notable observation. BBOEHEP has previously been identified as an oxidative transformation product of tris(2-butoxyethyl) phosphate (TBOEP), a widely used plasticizer and flame retardant in flooring materials, coatings, and polymer-based products. In the present dataset, the spatial distribution of BBOEHEP cannot be entirely attributed to single direct industrial emission sources, yet alternative sources cannot be confirmed by current data. Its occurrence across multiple indoor microenvironments, particularly those characterized by high densities of electronic devices and polymer-containing materials, is broadly consistent with the possibility of secondary formation processes, although direct mechanistic confirmation remains beyond the scope of the present study [41].

The present dataset does not allow direct discrimination between in situ formation and direct emission from commercial products. Although BBOEHEP and TBOEP were simultaneously detected, such correlational coexistence only matches the reaction routes summarized in prior laboratory studies and cannot prove causal oxidation reactions indoors [53]. Such pathways typically involve radical-mediated oxidation of alkoxy side chains, leading to the formation of oxygenated derivatives with modified physicochemical properties [54]. In this context, the synchronous detection of BBOEHEP and TBOEP merely implies a theoretical potential for indoor transformation, and cannot serve as credible evidence of continuous in situ reactions in collected dust samples.

Beyond this specific case, additional correlations only hint at the theoretical possibility that additive chemicals may undergo concurrent transformation processes within indoor environments. The most pronounced example is the co-occurrence of AO168 and its oxidation product AO168=O, a transformation pathway previously reported as a characteristic aging process of phosphite antioxidants in indoor dust systems [41]. The simultaneous presence of parent antioxidant and oxidation product suggests that oxidative processes may occur during material aging, and provides indirect evidence for the progressive transformation of additive-derived chemicals within indoor reservoirs [55,56]. However, the relative contribution of direct emission versus post-emission transformation remains unresolved based on the present observational dataset.

A broader implication of these observations is that indoor environments should increasingly be viewed as chemically active systems. Although reaction rates are generally slower than those observed outdoors, the combination of long residence times, abundant surface area, oxidant exposure, and continuous emissions may permit gradual transformation over months or years. From an exposure perspective, this distinction is critical because transformation products are not necessarily less hazardous than their parent compounds. In some cases, oxidation and dealkylation can increase biological activity by generating compounds with greater bioavailability or stronger receptor interactions.

### 3.5. Exposure Implications and Population Vulnerability

**Figure 5:**
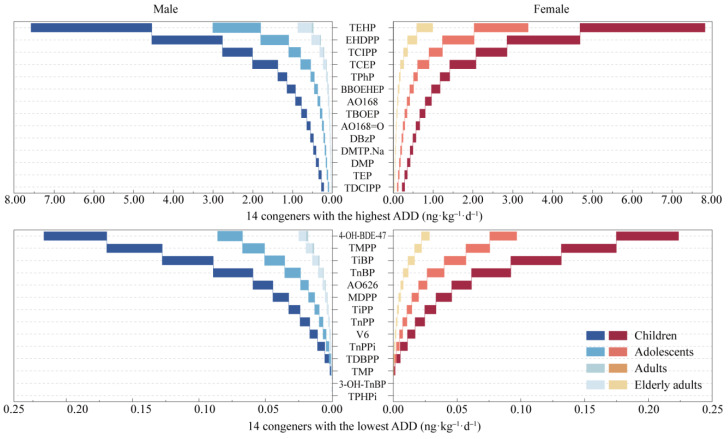
Comparison of ADD from Hand-to-Mouth Exposure to OPEs Among Different Gender and Age Groups.

**Figure 6:**
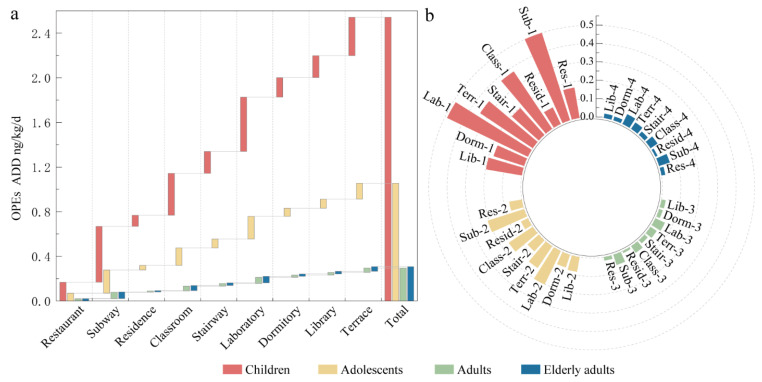
Exposure doses of OPEs in populations of different age groups. (**a**) ADD values in each microenvironment and total exposure doses; (**b**) Proportional contributions of different microenvironments to the total OPEs exposure in each age group.

The integrated exposure assessment revealed that settled dust ingestion is the predominant route of OPE intake across all age and gender groups, contributing over 98% of total exposure (Figure 5 and Figure 6). This dominance reflects the strong particle-phase affinity of high-molecular-weight and hydrophobic OPEs, including TEHP and EHDPP, which preferentially accumulate in indoor dust reservoirs. In contrast, inhalation and dermal absorption contributed minimally to overall exposure, highlighting the critical role of dust reservoirs in mediating chronic indoor exposure.

Age- and gender-specific analyses demonstrated a pronounced vulnerability of preschool children (3–6 years), whose total exposure via dust ingestion was approximately 2–3 times higher than that of adolescents and up to 9 times higher than that of adults. The elevated exposure in children arises from a combination of higher hand-to-mouth contact frequency, increased relative ingestion rates, and lower body mass, consistent with previous biomonitoring studies. Females exhibited slightly higher exposures than males within the same age group, largely attributable to lower body weight rather than differential behavior patterns.

Compound-level evaluation indicated that EHDPP, TEHP, TCIPP, and TCEP were the primary contributors to cumulative hazard, collectively accounting for more than 70% of non-carcinogenic risk metrics. EHDPP alone represented approximately 31% of total HI, reflecting both its high abundance and strong partitioning into dust. The presence of transformation products, such as BBOEHEP and AO168-O, introduces additional complexity. These compounds may exhibit altered bioavailability, enhanced receptor interactions, or increased endocrine-disrupting potential relative to their parent triesters, thereby influencing overall exposure–toxicity relationships in ways that conventional HI/CR calculations fail to capture.

To address these limitations, risk evaluation was framed within an exposure–transformation–toxicity continuum, integrating parent compounds, dust partitioning dynamics, and potential formation of bioactive transformation products. Within this framework, the cumulative exposure dose reflects not only the magnitude of primary emissions but also the environmental retention and chemical aging of OPEs within dust reservoirs. Transformation products such as BBOEHEP are of particular concern due to their persistence and emerging evidence of higher biological activity, implying that traditional assessments based solely on parent OPEs underestimate the true hazard potential in sensitive populations.

Spatially, the highest exposure doses were consistently associated with subway stations, laboratories, and classrooms, mirroring the distribution of dominant OPEs and reinforcing the link between material-rich microenvironments and population vulnerability. Even outdoor reference sites exhibited measurable exposure, although levels were substantially lower, confirming that indoor reservoirs amplify exposure risk relative to ambient background levels. The continuum framework also highlights that the toxicity potential of indoor OPEs is modulated by physicochemical properties and environmental partitioning. High-molecular-weight aryl phosphates, such as EHDPP, and secondary oxidation products exhibit low volatility and strong dust affinity, extending residence time and enhancing cumulative intake. This mechanism-driven perspective provides a more comprehensive understanding of why certain compounds disproportionately drive exposure and risk despite relatively low emission rates, emphasizing the need for mitigation strategies that target both material management and dust control in high-risk microenvironments.

These findings underscore that effective assessment of indoor OPE-associated health risks requires integration across three dimensions: emission source strength, environmental transformation and reservoir behavior, and compound-specific bioactivity. Children represent the most sensitive subpopulation due to higher relative intake and physiological susceptibility, while the disproportionate contribution of replacement OPEs and transformation products highlights the evolving nature of indoor chemical hazards. The exposure–transformation–toxicity continuum therefore provides a mechanistic scaffold for future monitoring, exposure modeling, and risk management strategies aimed at reducing human health impacts from emerging indoor contaminants.

One-way ANOVA combined with Tukey’s HSD post hoc test was performed on log_10_-transformed total OPE ADD of ingestion, inhalation and dermal pathways across four age subgroups. Full descriptive statistics, ANOVA summary tables, Levene’s homogeneity test and pairwise Tukey comparison results are provided in Tables S12–S14, respectively. Levene’s test confirmed significant heterogeneity of variances for all three exposure routes (all *p* < 0.001). For dominant hand-to-mouth ingestion and dermal exposure, significant age disparities were detected (*p* < 0.001). Tukey pairwise comparisons demonstrated that toddlers exhibited markedly higher exposure doses than teenagers, adults and elderly adults, while no statistical gap existed between adults and the elderly. In contrast, inhalation exposure showed a distinct pattern: only teenagers had significantly lower ADD compared with other age cohorts, and no significant difference was observed between toddlers, adults and elderly adults.

Taken together, children exhibited markedly higher OPE exposure risks via dust ingestion than other age groups. It should be noted that the above risk assessment was conducted via deterministic calculation without uncertainty simulation, and dust OPE bioaccessibility was not corrected during dose estimation. In addition, the additive HQ and HI models cannot reflect mixture toxic interactions and the toxicity of indoor transformation products. Relevant optimization including Monte Carlo simulation, bioaccessibility correction and mixture toxicity evaluation will be implemented in follow-up studies.

## 4. Conclusions

Indoor dust acts as an integrative medium reflecting continuous emissions, phase partitioning, and potential chemical aging of organophosphate esters. The dominance of TEHP and EHDPP suggests that alternative flame retardants tend to be persistently incorporated in indoor chemical systems. Evidence for transformation products such as BBOEHEP and AO168=O indicates that indoor environments may also support slow oxidative processing, although the extent and kinetics of these reactions remain uncertain. Exposure is primarily driven by dust ingestion, with children representing a more sensitive population due to behavioral and physiological factors. However, the correlation analysis and PMF source apportionment applied herein carry inherent analytical constraints, and current risk estimates are constrained by incomplete understanding of transformation products and bioaccessibility. Future work should integrate non-target chemical analysis, transformation kinetics, bioaccessibility assessment, and mechanism-based toxicity assessment to better resolve the coupling between indoor chemical dynamics and human exposure.

## Data Availability

The data presented in this study are available within the article and its Supplementary Materials. Additional data supporting the findings of this study are available from the corresponding author upon reasonable request.

## Supplementary Materials

The following supporting information can be downloaded at https://www.mdpi.com/article/10.3390/toxics14070629/s1, Text S1: Detailed Specifications for Instrumental Analysis; Text S2: Data Preprocessing and Calculation Methods for PMF Model; Text S3: Introduction to the ADD Model and Computational Methods; Text S4: Calculation Methods for HQ, HI and CR; Table S1: Target OPEs and Quantifier/Qualifier Ions for Instrumental Determination; Table S2: Correlation Coefficient of the Target OPEs Standard Curve; Table S3: Background Concentrations, Limits of Detection, Limits of Quantification, Precision and Spike Recoveries of Target OPEs; Table S4: Parameters Used for Exposure Assessment; Table S5: Non-carcinogenic Reference Dose (RfD) and CSF of OPEs; Table S6: Summary of Mean, Standard Deviation (SD), Standard Error (SE) and 95% Two-Sided Confidence Interval (95% CI) for log_10_-Transformed OPE Concentrations from Nine Indoor Sampling Sites; Table S7: Results of One-Way ANOVA and Levene’s Homogeneity of Variance Test for log_10_-Transformed Mixed OPE Concentrations Across Nine Indoor Sampling Sites; Table S8: Full Pairwise Comparison Outputs of Tukey’s HSD Post Hoc Test; Table S9: Q Values of All Convergent PMF Model Runs; Table S10: Mapping Rates of Bootstrap Factors to Base PMF Source Factors; Table S11: Concentrations of Target Compounds across Indoor Functional Environments (mg·kg^−1^); Table S12: Summary of Mean, SD, SE and 95% CI for log_10_-Transformed Total OPE ADD via Ingestion, Inhalation and Dermal Pathways across Four Age Subgroups; Table S13: Results of One-Way ANOVA and Levene’s Homogeneity of Variance Test for log_10_-Transformed Total OPE ADD of Three Exposure Pathways among Four Age Subgroups; Table S14: Full Pairwise Comparison Outputs of Tukey’s HSD Post Hoc Test for Age-Stratified log_10_-Transformed Total OPE ADD via Three Exposure Routes; Figure S1: Boxplots of Log_10_-Transformed Mixed OPE Concentrations and Scaled Residual Histogram of PMF Model.

## References

[1] Castro-Jiménez J., González-Gaya B., Pizarro M., Casal P., Pizarro-Álvarez C., Dachs J. (2016). Organophosphate Ester Flame Retardants and Plasticizers in the Global Oceanic Atmosphere. Environ. Sci. Technol..

[2] Liu Q., Liu R., Zhang X., Li W., Harner T., Saini A., Liu H., Yue F., Zeng L., Zhu Y. (2023). Oxidation of commercial antioxidants is driving increasing atmospheric abundance of organophosphate esters: Implication for global regulation. One Earth.

[3] Liu B., Ding L., Lv L., Yu Y., Dong W. (2023). Organophosphate esters (OPEs) and novel brominated flame retardants (NBFRs) in indoor dust: A systematic review on concentration, spatial distribution, sources, and human exposure. Chemosphere.

[4] Azizi S., Dehghani M.H., Naddafi K., Nabizadeh R., Yunesian M. (2023). Occurrence of organophosphorus esters in outdoor air fine particulate matter and comprehensive assessment of human exposure: A global systematic review. Environ. Pollut..

[5] Zhang Q., Yang L., Wang H., Wu C., Cao R., Zhao M., Su G., Wang C. (2024). A comprehensive evaluation of the endocrine-disrupting effects of emerging organophosphate esters. Environ. Int..

[6] He W., Ding J., Gao N., Zhu L., Zhu L., Feng J. (2024). Elucidating the toxicity mechanisms of organophosphate esters by adverse outcome pathway network. Arch. Toxicol..

[7] Xie Y., Peng R., Xiao L. (2025). Environmental Chemicals and Female Reproductive Health: Unraveling Mechanisms and Societal Impacts—A Narrative Review. Clin. Exp. Obstet. Gynecol..

[8] Guo Y., Chen M., Liao M., Su S., Sun W., Gan Z. (2023). Organophosphorus flame retardants and their metabolites in paired human blood and urine. Ecotoxicol. Environ. Saf..

[9] Gao D.T., Yang J., Bekele T.G., Zhao S.J., Zhao H.X., Li J., Wang M.J., Zhao H.D. (2020). Organophosphate esters in human serum in Bohai Bay, North China. Environ. Sci. Pollut. Res..

[10] Hou M.M., Shi Y.L., Jin Q., Cai Y.Q. (2020). Organophosphate esters and their metabolites in paired human whole blood, serum, and urine as biomarkers of exposure. Environ. Int..

[11] Qiao L., Zheng X.B., Zheng J., Chen S.J., Zhong C.Q., Chen J.H., Yang Z.Y., Mai B.X. (2019). Legacy and Currently Used Organic Contaminants in Human Hair and Hand Wipes of Female E-Waste Dismantling Workers and Workplace Dust in South China. Environ. Sci. Technol..

[12] Zheng G., Schreder E., Dempsey J.C., Uding N., Chu V., Andres G., Sathyanarayana S., Salamova A. (2021). Organophosphate Esters and Their Metabolites in Breast Milk from the United States: Breastfeeding Is an Important Exposure Pathway for Infants. Environ. Sci. Technol. Lett..

[13] Hammel S.C., Zhang S., Lorenzo A.M., Eichner B., Stapleton H.M., Hoffman K. (2020). Young infants’ exposure to organophosphate esters: Breast milk as a potential source of exposure. Environ. Int..

[14] Hou R., Xu Y., Wang Z. (2016). Review of OPFRs in animals and humans: Absorption, bioaccumulation, metabolism, and internal exposure research. Chemosphere.

[15] Hong Z.J., Man Y.B., Li Y., Xiao L.Y., Han S.T., Chen Z.Y., Lan B.Y., Yan X.M., Luo J.W., Zeng L.X. (2025). Dermal bioaccessibility of organophosphate esters in indoor dust: Influencing factors, release kinetics, and health risk assessment. J. Hazard. Mater..

[16] Cai Y.M., Xu M.Y., Ouyang M.H., Wu Y.S., Wang R.J., Zheng K.W., Ren G.F. (2025). Concentrations, Compositions and Human Exposure Risks to Organophosphate Esters in Indoor Air from Various Microenvironments in Guangzhou, China. Toxics.

[17] Solanke A., Talalaj L., Graham C., Alegria H. (2025). Organophosphate Flame Retardants in Indoor Dust in the Tampa Bay (Florida) Area. Toxics.

[18] Dong X.Y., Pei J.J., Qu M.N. (2025). Organophosphate esters (OPEs) in the air and dust of new vehicle cabins: Concentrations, sources and contributions. Build. Environ..

[19] Foster S.A., Kile M.L., Hystad P., Diamond M.L., Mandhane P.J., Moraes T.J., Pei J., Scott J.A., Simons E., Subbarao P. (2026). Organophosphate ester flame retardants and plasticizers in house dust and Child Behavior Checklist outcomes: A nested study in the Canadian CHILD Birth Cohort. J. Expo. Sci. Environ. Epidemiol..

[20] Persson J., Hagberg J., Carlberg M., Wang T. (2025). Children’s exposure risk toward brominated flame retardants and organophosphate esters during preschool attendance and potential contamination sources. Int. J. Hyg. Environ. Health.

[21] Chen Y., Zhang Q., Luo T., Xing L., Xu H. (2019). Occurrence, distribution and health risk assessment of organophosphate esters in outdoor dust in Nanjing, China: Urban vs. rural areas. Chemosphere.

[22] Duan X.L. (2016). Exposure Factors Handbook of Chinese Population (Children).

[23] Duan X.L. (2013). Exposure Factors Handbook of Chinese Population (Adults).

[24] General Administration of Sport of China The 6th National Physical Fitness Monitoring Bulletin. https://www.sport.gov.cn/n315/n20001395/c29322125/content.html.

[25] Ministry of Ecology and Environment of the People’s Republic of China Technical Guidelines for Risk Assessment of Soil Contamination of Land for Construction. https://www.mee.gov.cn/ywgz/fgbz/bz/bzwb/trhj/201912/t20191224_749893.shtml.

[26] General Administration of Sport of China 2014 National Physical Fitness Monitoring Bulletin. https://www.sport.gov.cn/n315/n329/c216784/content.html.

[27] US Environmental Protection Agency The Risk Assessment Information System. https://rais.ornl.gov/cgi-bin/tools/TOX_search?select=chemtox.

[28] Wignall J.A., Muratov E., Sedykh A., Guyton K.Z., Tropsha A., Rusyn I., Chiu W.A. (2018). Conditional toxicity value (CTV) predictor: An in silico approach for generating quantitative risk estimates for chemicals. Environ. Health Perspect..

[29] Lian M.S., Lin C.Y., Li Y., Hao X., Wang A.H., He M.C., Liu X.T., Ouyang W. (2022). Distribution, partitioning, and health risk assessment of organophosphate esters in a major tributary of middle Yangtze River using Monte Carlo simulation. Water Res..

[30] Lv J.P., Guo C.S., Luo Y., Liu Y., Deng Y.H., Sun S.W., Xu J. (2022). Spatial distribution, receptor modelling and risk assessment of organophosphate esters in surface water from the largest freshwater lake in China. Ecotoxicol. Environ. Saf..

[31] Li X.H., Tian T., Shang X.C., Zhang R.H., Xie H.J., Wang X.J., Wang H.W., Xie Q., Chen J.W., Kadokami K. (2020). Occurrence and Health Risks of Organic Micro-Pollutants and Metals in Groundwater of Chinese Rural Areas. Environ. Health Perspect..

[32] European Parliament and the Council of the European Union Regulation (EC) No 1272/2008 on Classification, Labelling and Packaging of Substances and Mixtures. https://eur-lex.europa.eu/legal-content/EN/TXT/PDF/?uri=CELEX:32008R1272.

[33] International Agency for Research on Cancer (IARC) Agents Classés par les Monographies du CIRC, Volumes 1–138. https://monographs.iarc.who.int/fr/agents-classes-par-les-monographies-du-circ-2/.

[34] Kademoglou K., Xu F.C., Padilla-Sanchez J.A., Haug L.S., Covaci A., Collins C.D. (2017). Legacy and alternative flame retardants in Norwegian and UK indoor environment: Implications of human exposure via dust ingestion. Environ. Int..

[35] Yadav I.C., Devi N.L., Zhong G., Li J., Zhang G., Covaci A. (2017). Occurrence and fate of organophosphate ester flame retardants and plasticizers in indoor air and dust of Nepal: Implication for human exposure. Environ. Pollut..

[36] Li W., Wang Y., Asimakopoulos A.G., Covaci A., Gevao B., Johnson-Restrepo B., Kumosani T.A., Malarvannan G., Moon H.-B., Nakata H. (2019). Organophosphate esters in indoor dust from 12 countries: Concentrations, composition profiles, and human exposure. Environ. Int..

[37] Tan H.L., Yang L., Yu Y.J., Guan Q.X., Liu X.T., Li L.Z., Chen D. (2019). Co-Existence of Organophosphate Di- and Tri-Esters in House Dust from South China and Midwestern United States: Implications for Human Exposure. Environ. Sci. Technol..

[38] Kim U.-J., Wang Y., Li W., Kannan K. (2019). Occurrence of and human exposure to organophosphate flame retardants/plasticizers in indoor air and dust from various microenvironments in the United States. Environ. Int..

[39] Vykoukalová M., Venier M., Vojta Š., Melymuk L., Bečanová J., Romanak K., Prokeš R., Okeme J.O., Saini A., Diamond M.L. (2017). Organophosphate esters flame retardants in the indoor environment. Environ. Int..

[40] Hartmann P.C., Bürgi D., Giger W. (2004). Organophosphate flame retardants and plasticizers in indoor air. Chemosphere.

[41] Liu R., Mabury S.A. (2019). Organophosphite Antioxidants in Indoor Dust Represent an Indirect Source of Organophosphate Esters. Environ. Sci. Technol..

[42] Cristale J., Hurtado A., Gómez-Canela C., Lacorte S. (2016). Occurrence and sources of brominated and organophosphorus flame retardants in dust from different indoor environments in Barcelona, Spain. Environ. Res..

[43] Wong F., de Wit C.A., Newton S.R. (2018). Concentrations and variability of organophosphate esters, halogenated flame retardants, and polybrominated diphenyl ethers in indoor and outdoor air in Stockholm, Sweden. Environ. Pollut..

[44] He C., Wang X., Thai P., Baduel C., Gallen C., Banks A., Bainton P., English K., Mueller J.F. (2018). Organophosphate and brominated flame retardants in Australian indoor environments: Levels, sources, and preliminary assessment of human exposure. Environ. Pollut..

[45] Cristale J., Aragão Belé T.G., Lacorte S., Rodrigues de Marchi M.R. (2018). Occurrence and human exposure to brominated and organophosphorus flame retardants via indoor dust in a Brazilian city. Environ. Pollut..

[46] Abafe O.A., Martincigh B.S. (2019). Concentrations, sources and human exposure implications of organophosphate esters in indoor dust from South Africa. Chemosphere.

[47] Wu Y.L., Liu S.Q., Dong H., Zhang J.M., Lu R., Yang Q.Y., Jaman R., Zhu J.M., Zhang C.L., Zhou J.Q. (2026). Environmental occurrence, human exposure and multidimensional toxicity effects of 2-ethylhexyl diphenyl phosphate (EHDPP): Toxicological mechanism analysis and future research prospects. Ecotoxicol. Environ. Saf..

[48] Lao J.-Y., Huang G., Wu R., Liang W., Xu S., Luo Q., Zhang K., Jing L., Jin L., Ruan Y. (2024). Aggravating Pollution of Emerging Aryl Organophosphate Esters in Urban Estuarine Sediments of South China. Environ. Sci. Technol..

[49] Hou M., Shi Y., Na G., Cai Y. (2021). A review of organophosphate esters in indoor dust, air, hand wipes and silicone wristbands: Implications for human exposure. Environ. Int..

[50] Liang Y., Liu X., Allen M.R. (2018). Measurements of Parameters Controlling the Emissions of Organophosphate Flame Retardants in Indoor Environments. Environ. Sci. Technol..

[51] Song X., Zhu S., Hu L., Chen X., Zhang J., Liu Y., Bu Q., Ma Y. (2024). A Review of the Distribution and Health Effect of Organophosphorus Flame Retardants in Indoor Environments. Toxics.

[52] Lao J.Y., Lin H., Qin X., Ruan Y., Leung K.M.Y., Zeng E.Y., Lam P.K.S. (2022). Insights into the Atmospheric Persistence, Transformation, and Health Implications of Organophosphate Esters in Urban Ambient Air. Environ. Sci. Technol..

[53] Wang S., Jin J., Ma Y., Stubbings W.A., Gbadamosi M.R., Abou-Elwafa Abdallah M., Harrad S. (2024). Organophosphate triesters and their diester degradation products in the atmosphere—A critical review. Environ. Pollut..

[54] Huo Y., Li M., Jiang J., Zhou Y., Ma Y., Xie J., He M. (2023). The aomogeneous and heterogeneous oxidation of organophosphate esters (OPEs) in the atmosphere: Take diphenyl phosphate (DPhP) as an example. Environ. Pollut..

[55] Liu R., Lin Y., Ruan T., Jiang G. (2017). Occurrence of synthetic phenolic antioxidants and transformation products in urban and rural indoor dust. Environ. Pollut..

[56] Liu X., Chen D., Yu Y., Zeng X., Li L., Xie Q., Yang M., Wu Q., Dong G. (2020). Novel Organophosphate Esters in Airborne Particulate Matters: Occurrences, Precursors, and Selected Transformation Products. Environ. Sci. Technol..

