# Supplementary material for: Spatial Distribution Characteristics and Human Health Risk Assessment of Organophosphate Esters in Indoor Dust in Beijing
Source: Toxics. 2026 Jul 20;14(7):629. doi: 10.3390/toxics14070629 (PMC13419270; doi:10.3390/toxics14070629)
Supplement: Supplementary file 1 [file toxics-14-00629-s001.zip › toxics-4408199-supplementary.pdf]

Supplementary

Spatial Distribution Characteristics and Human Health Risk Assessment of Organophosphate Esters in Indoor Dust in Beijing

Huizi Yuan [1], Ziyan Chen [2], Weicheng Zhao [2], Mingyang Li [1], Yinghong Wang [3], Xingru Li [2,*] and Fangkun Wu [3,*]

1. College of Resource Environment and Tourism, Capital Normal University, Beijing 100048, China

2. Analysis and Testing Center, Department of Chemistry, Capital Normal University, Beijing 100048, China

3. Institute of Atmospheric Physics, Chinese Academy of Sciences, Beijing 100029, China

Correspondence: lixr@cnu.edu.cn (X.L.); wfk@mail.iap.ac.cn (F.W.)

Text S1: Detailed Specifications for Instrumental Analysis

Text S2: Data Preprocessing and Calculation Methods for PMF Model

Text S3: Introduction to the ADD Model and Computational Methods

Text S4: Calculation Methods for HQ, HI and CR

Table S1: Target OPEs and Quantifier/Qualifier Ions for Instrumental Determination

Table S2: Correlation Coefficient of the Target OPEs Standard Curve

Table S3: Background Concentrations, Limits of Detection, Limits of Quantification, Precision and Spike Recoveries of Target OPEs

Table S4: Parameters Used for Exposure Assessment

Table S5: Non-carcinogenic Reference Dose (RfD) and CSF of OPEs

Table S6: Summary of Mean, Standard Deviation (SD), Standard Error (SE) and 95% Two-Sided Confidence Interval (95% CI) for $\log_{10}$-Transformed OPE Concentrations from Nine Indoor Sampling Sites

Table S7: Results of One-Way ANOVA and Levene's Homogeneity of Variance Test for $\log_{10}$-Transformed Mixed OPE Concentrations Across Nine Indoor Sampling Sites

Table S8: Full Pairwise Comparison Outputs of Tukey's HSD Post-Hoc Test

Table S9: Q Values of All Convergent PMF Model Runs

Table S10: Mapping Rates of Bootstrap Factors to Base PMF Source Factors

Table S11: Concentrations of Target Compounds across Indoor Functional Environments ($mg \cdot kg^{-1}$)

Table S12: Summary of Mean, SD, SE and 95% CI for $\log_{10}$-Transformed Total OPE ADD via Ingestion, Inhalation and Dermal Pathways across Four Age Subgroups

Table S13: Results of One-Way ANOVA and Levene's Homogeneity of Variance Test for log_{10}-Transformed Total OPE ADD of Three Exposure Pathways among Four Age Subgroups

Table S14: Full Pairwise Comparison Outputs of Tukey's HSD Post-Hoc Test for Age-Stratified log_{10}-Transformed Total OPE ADD via Three Exposure Routes

Figure S1: Boxplots of Log_{10}-Transformed Mixed OPE Concentrations and Scaled Residual Histogram of PMF Model

Text S1: Detailed Specifications for Instrumental Analysis

Qualitative and quantitative analyses of the sample extracts were performed using ultra-high-performance liquid chromatography (ACQUITY UPLC, Waters, USA) and high-resolution mass spectrometry with a triple quadrupole linear ion trap (6500 QTRAP, SCIEX, USA). Text S1 Chromatographic separation was performed using a reverse-phase column (F5100 mm × 3.0 mm × 2.6 μm, 1000 nm, Phenomenex, USA). Mobile phase A consisted of an aqueous solution containing 0.1 mM formic acid (LC-MS, Fisher Scientific, USA), and mobile phase B was acetonitrile. The flow rate was 0.35 mL·min^{-1}, and the column temperature was 40°C. The mobile phase gradient elution program was set as follows: the initial condition was 10% B and 90% A; this was held constant for 0.20 min; then linearly increased to 70% B by 9.00 min; further increased to 98% B at 10.00 min and held until 14.50 min; rapidly returned to the initial conditions (10% B) at 15.00 min; and maintained at 10% B until the end of the run at 25.00 min to achieve system equilibrium. The ion source was electron spray ionization (ESI); Detection method: Multiple Reaction Monitoring (MRM); Curtain gas (CUR): 20 psi; Ion source gas: 50 psi; Collision gas flow rate: 50 psi; Ion spray voltage (IS): 5500 V; Ion source temperature (Tem): 500°C.

Text S2: Data Preprocessing and Calculation Methods for PMF Model

This study employed Positive Matrix Factorization (PMF) using EPA PMF 5.0 to analyze the potential sources of 28 OPEs in eight types of indoor dust based on concentration data and uncertainty data for each species in the samples. The concentration data for each substance were obtained experimentally, and the general method for calculating uncertainty (Unc) is as follows. If the concentration of a substance is less than or equal to the provided minimum detectable limit (MDL), the uncertainty is calculated as a fixed proportion of the MDL, as shown in Equation (S1).

$$Unc = MDL \times \frac{5}{6} \quad (S1)$$

Here, MDL stands for Method Detection Limit.

If the concentration of a substance exceeds the specified MDL, the calculation will be based on the concentration fraction of the substance and the MDL, as shown in Equation (S2).

$$Unc = \sqrt{(RSD\_m \times c)^2 + (0.5 \times MDL)^2} \quad (S2)$$

In the equation, RSD_m represents the accuracy of the substance, in percent; *c* represents the average concentration of OPEs in indoor dust, in $ng \cdot mg^{-1}$.

Text S3: Introduction to the ADD Model and Computational Methods

Based on the carcinogen classification lists issued by the EU CLP Regulation (Regulation on Classification, Labelling and Packaging of Substances and Mixtures, hereinafter referred to as EU CLP) and the International Agency for Research on Cancer (IARC), six out of the 28 detected OPEs, including TCIPP, TCEP and TDCIPP, were classified as carcinogens or probable carcinogens. Detailed information is presented in Table S5.

Based on the concentration of OPEs in indoor environments, the potential human exposure dose is calculated using the ADD model provided by the U.S. Environmental Protection Agency (EPA), as shown in the following formula:

$$ADD_{oral} = C \times \frac{IR_{oral} \times EF_{oral} \times ED_{oral}}{BW \times AT} \quad (S3)$$

$$ADD_{inh} = c \times \frac{IR_{inh} \times EF_{inh} \times ED_{inh}}{PEF \times BW \times AT} \quad (S4)$$

$$ADD_{dermal} = c \times \frac{SA \times SL \times ABS \times EF_{dermal} \times ED_{dermal}}{BW \times AT} \quad (S5)$$

In this context, ADD_{oral}, ADD_{inh}, and ADD_{der} represent the average daily exposure levels via the hand-to-mouth route, inhalation, and skin contact, respectively, in $mg \cdot kg^{-1} \cdot day^{-1}$; *c* is the average concentration of OPEs in indoor dust, in $ng \cdot mg^{-1}$; IR_{oral} is the rate of OPEs intake via the hand-to-mouth route, in $mg \cdot day^{-1}$; IR_{inh} is the rate of OPEs intake via the inhalation route, in $m^3 \cdot day^{-1}$; BW is the average body weight, kg; EF is the annual exposure frequency for the three routes, $day \cdot a^{-1}$; ED is the number of exposure years, a; AT_{nc} is the averaging time for non-carcinogenic risk assessment, day; AT_c is the averaging time for carcinogenic risk assessment, day; PEF is the particulate matter emission factor, $m^3 \cdot kg^{-1}$; SA is the exposed skin area, m^2; SL is the skin adhesion coefficient, $mg \cdot (cm^2 \cdot d)^{-1}$; ABS is the skin absorption coefficient. The meanings and values of other parameters in the formula are shown in Table S4.

The risk assessment in this study utilized localized parameters for certain exposure factors (including intake rate, average body weight, average exposure duration, exposed skin area, and skin adhesion coefficient), which helps identify and protect vulnerable groups within specific subpopulations in China (such as children and the elderly) and provides a scientific basis for developing indoor environmental quality standards and health protection strategies tailored to the country's specific conditions.

Text S4: Calculation Methods for HQ, HI and CR

Based on the ADD of OPEs, this study employed the Hazard Quotient (HQ) to assess the health risks associated with the target population's exposure to OPEs in indoor dust. When $HQ < 1$, it indicates that the pollutant poses no unexpected adverse health effects to humans. The Hazard Index (HI) is used to characterize the combined non-carcinogenic health risks of multiple substances; when $HI < 1$, the risk is considered acceptable; when $HI \geq 1$, there is a potential non-

carcinogenic health risk. The calculation methods for HQ and HI are shown in Equations (S6) and (S7).

$$HQ=\frac{ADD}{RfD} \tag{S6}$$

$$HI=\sum HQ \tag{S7}$$

In the equation, RfD represents the reference dose (ng·kg^{-1}·day^{-1}). The relevant parameters are based on the EPA Integrated Risk Information System (IRIS) and Chen et al. [21]; see Table S5.

In this study, the carcinogenic risk (CR) assessment was conducted for five OPEs with established cancer slope factors (CSFs), including TEHP, TCEP, and TDCIPP. Such substances are either classified as carcinogens or suspected carcinogens by authoritative bodies (such as IARC and the EU CLP Regulation), or have CSF values derived based on existing experimental evidence. In accordance with the precautionary principle in risk assessment, a quantitative evaluation of their potential lifetime carcinogenic health effects was performed. For all OPEs with CSF values, CR was calculated as the product of the Lifetime Average Daily Dose (LADD) and the CSF, using the following formula:

$$CR=LADD\times CSF \tag{S8}$$

In the equation, LADD represents the lifetime average daily dose of OPEs, calculated as shown in Equations (S3) through (S5); the values of the relevant parameters are listed in Table S4; CSF is the carcinogenic effect slope factor for each organic pollutant (ng·kg^{-1}·day^{-1}); relevant parameter values are shown in Table S5. A CR value of $< 1.0\times10^{-6}$ indicates a negligible cancer risk, a value between 1.0×10^{-6} and 1.0×10^{-4} indicates a potential cancer risk, and a CR value $> 1.0\times10^{-4}$ indicates a high cancer risk.

Table S1: Target OPEs and Quantifier/Qualifier Ions for Instrumental Determination

Abbr.	CAS	Precursor ion	Product ion	DP / V	CE
TEHP	78-42-2	435.2	99	75	18
EHDPP	1241-94-7	363.3	251.4	75	40
TCIPP	13674-84-5	327.1	99	63	24
TCEP	115-96-8	285	62.9	78	37
TPhP	115-86-6	327.1	77	109	51
TBOEP	78-51-3	399.4	199.1	70	23
TDCIPP	13674-87-8	430.9	98.7	75	38
TEP	78-40-0	183.1	81	50	46
TMPP	1330-78-5	369.4	91	120	47
TiBP	126-71-6	267.2	99	52	21
TnBP	126-73-8	267.2	155	52	12
TnPP	513-08-6	225.1	141	50	13
TiPP	513-02-0	225.1	99	50	19
V6	38051-10-4	583	360.9	94	27
TDBPP	126-72-7	698.5	98.6	87	49
TMP	512-56-1	141.1	109	63	29
MDPP	115-89-9	265	93	45	17
3-OH-TnBP	89197-69-3	283	99	20	20
DBzP	1623-08-1	227	79	33	33
TPHPi	101-02-0	311.1	217.2	40	18
AO168	31570-04-4	647.4	234.9	69	60

AO626	26741-53-7	605.3	279.2	56	24
TnPPi	26523-78-4	689.3	91.1	40	58
AO168=O	95906-11-9	663.3	495.3	75	40
4-OH-BDE-47	56806-74-7	343	77	55	22
BBOEHEP	1477494-86-2	343.2	243	100	6
DMP	813-78-5	125	63	90	24
DMTP.Na	23754-87-2	141	79	32	17

Table S2: Correlation Coefficient of the Target OPEs Standard Curve

Abbr.	k	b	R^2
TEHP	1364033	-70307	0.9966
EHDPP	7759768	-1301884	0.9872
TCIPP	190739	83653	0.9911
TCEP	18185200	489208	0.9985
TPhP	57916800	-613299	0.9976
TBOEP	839226000	-30051400	0.9986
TDCIPP	21895300	-374082	0.996
TEP	80642000	2080679	0.9987
TMPP	58909200	5780936	0.9907
TiBP	978722000	-8377313	0.9988
TnBP	646786000	-17115200	0.9998
TnPP	557483000	-9336010	0.9979
TiPP	539063000	-3816599	0.9959
V6	60741000	-3145524	0.9909
TDBPP	883132	1301	0.9696
TMP	120078000	2939089	0.9982
MDPP	39686	3726.617	0.9999
3-OH-TnBP	268000000	-3892456	0.9999
DBzP	27990000	-107647	0.9989
TPHPi	97486200	-5092906	0.9995
AO168	3476175	50382	0.9937

AO626	2685137	33694	0.994
TnPPi	1226818	-210478	0.9905
AO168=O	110000000	339787	0.9982
4-OH-BDE-47	190739	83653	0.9384
BBOEHEP	5955	310	0.9926
DMP	11538	2300	0.9989
DMTP.Na	35942	9393.977	0.9999

Table S3: Background Concentrations, Limits of Detection, Limits of Quantification, Precision and Spike Recoveries of Target OPEs

Abbr.	Blank / μg·mL^{-1}	LOD /μg·mL^{-1}	LOQ /μg·mL^{-1}	RSD_m / %	SR / %
TEHP	N.D.	0.1938	0.6460	4.64	103.57
EHDPP	N.D.	0.0617	0.2057	1.39	112.38
TCIPP	N.D.	0.0817	0.2723	4.23	85.29
TCEP	0.0013	0.0326	0.1087	10.21	94.57
TPhP	N.D.	0.0782	0.2607	6.45	108.37
TBOEP	N.D.	0.0518	0.1727	5.73	119.29
TDCIPP	0.0192	0.0575	0.1917	4.57	87.38
TEP	N.D.	0.0441	0.1470	5.05	91.38
TMPP	0.0079	0.1634	0.5447	7.84	96.74
TiBP	0.0454	0.3114	1.0380	1.04	102.94
TnBP	0.0454	0.0271	0.0903	5.66	80.00
TnPP	N.D.	0.0456	0.1522	2.24	88.12
TiPP	N.D.	0.0844	0.2813	4.23	117.29
V6	0.0071	0.0767	0.2557	0.62	92.12
TDBPP	N.D.	0.1524	0.5080	4.57	110.38
TMP	N.D.	0.1581	0.5271	0.24	83.74
MDPP	N.D.	0.0812	0.2707	0.55	89.29
3-OH-TnBP	N.D.	0.0617	0.2057	5.05	105.74
DBzP	0.0574	0.1305	0.435	4.23	98.38
TPHPi	0.0845	0.0369	0.123	2.24	115.29

AO168	0.0912	0.4681	1.5603	1.04	84.57
AO626	0.0345	0.2918	0.9727	5.66	107.12
TnPPi	0.0424	1.4323	4.7743	10.21	93.38
AO168=O	0.0081	0.4391	1.4637	6.45	100.29
4-OH-BDE-47	0.0741	0.0298	0.0993	4.27	118.74
BBOEHEP	0.1916	0.1555	0.5183	1.39	90.38
DMP	0.0116	0.0594	0.1980	7.84	104.29
DMTP.Na	0.0626	0.6351	2.117	5.73	111.57

Table S4: Parameters Used for Exposure Assessment

Parameter	Value							
	Children		Adolescents		Adults		Elderly adults	
	Male	Female	Male	Female	Male	Female	Male	Female
BW / kg	19.4[b]	18.8[b]	48.9[d]	43.5[d]	73.4[b]	59.6[b]	67.6[b]	59.4[b]
IRoral / $mg \cdot day^{-1}$	50[a]				20[a]			
IRinh / $m^3 \cdot day^{-1}$	7.6[a]				20[a]			
ED / a	6[c]				24[c]			
SA / m^2	0.28[a]				0.57[a]			
SL / $mg \cdot (cm^2 \cdot day)^{-1}$	0.2[a]				0.07[a]			
AT_{nc} / day	2190[a]				8760[a]			
AT_c / day	29265.7[a]							
PEF / $m^3 \cdot kg^{-1}$	1.36×10^9							
EF / $day \cdot a^{-1}$	45.88[c]							
ABS	0.001							

*Note:

a. These data are sourced from the Exposure factors handbook of Chinese population [22,23];

b. These data are sourced from the 6th National Physical Fitness Monitoring Bulletin of China (2025) [24];

c. These data are sourced from the Technical Guidelines for Risk Assessment of Contaminated Land (HJ 25.3—2019) [25].

d. This data is sourced from the 4th National Physical Fitness Monitoring Bulletin of China (2014) [26];

Table S5: Non-carcinogenic Reference Dose (RfD) and CSF of OPEs

**Abbr.**	**RfD / ng·kg^{-1}·day^{-1}**	**CSF / ng·kg^{-1}·day^{-1}**	**Carcinogenic Classification**
TEHP	100000[a]	3.0E-09[c]	/
EHDPP	12400[a]	/	/
TCIPP	10000[a]	/	Category 2[d]
TCEP	7000[a]	2.0E-08[c]	IARC Group 3[e]
TPhP	3670[a]	/	/
TBOEP	12000[b]	/	/
TDCIPP	20000[a]	3.1E-08[c]	Category 2[d]
TMPP	20000[b]	/	/
TnBP	10000[c]	9.0E-09[c]	Category 2[d]
TDBPP	/	/	IARC Group 2A[e]
TMP	10000[c]	2.0E-08[c]	Category 2[d]

*Notes:

a. Data obtained from the Risk Assessment Information System (RAIS) [27];

b. Data predicted by the QSAR model [28];

c. Data retrieved from published literature [29-31];

d. Data sourced from the EU CLP Regulation [32];

e. Data sourced from the International Agency for Research on Cancer (IARC) [33];

/. No available data.

Table S6: Summary of Mean, Standard Deviation (SD), Standard Error (SE) and 95% Two-Sided Confidence Interval (95% CI) for log_{10}-Transformed OPE Concentrations from Nine Indoor Sampling Sites

Sampling Site	Mean	SD	SE	Lower 95% CI	Upper 95% CI
Restaurant (Res.)	-8.57E-01	6.70E-01	1.27E-01	-6.09E-01	-1.11E+00
Subway (Sub.)	-7.17E-01	8.84E-01	1.67E-01	-3.89E-01	-1.04E+00
Residence (Resid.)	-1.06E+00	7.47E-01	1.41E-01	-7.82E-01	-1.34E+00
Classroom (Class.)	-8.61E-01	9.14E-01	1.73E-01	-5.23E-01	-1.20E+00
Stairway (Stair.)	-9.91E-01	7.97E-01	1.51E-01	-6.96E-01	-1.29E+00
Laboratory (Lab.)	-6.80E-01	8.14E-01	1.54E-01	-3.78E-01	-9.82E-01
Dormitory (Dorm.)	-9.56E-01	7.70E-01	1.46E-01	-6.71E-01	-1.24E+00
Library (Lib.)	-9.55E-01	7.91E-01	1.49E-01	-6.62E-01	-1.25E+00
Terrace (Terr.)	-6.98E-01	7.08E-01	1.34E-01	-4.36E-01	-9.60E-01

Table S7: Results of one-way ANOVA and Levene's homogeneity of variance test for log_{10}-transformed mixed OPE concentrations across nine indoor sampling sites.

		df	SS	MS	F	p-value
ANOVA test	Between groups	8	4.31E+00	5.39E-01	8.60E-01	5.51E-01
	Within groups	243	1.52E+02	6.27E-01		
Levene test	Between groups	8	1.21E+00	1.52E-01	6.77E-01	7.11E-01
	Within groups	243	5.44E+01	2.24E-01		

Table S8: Full pairwise comparison outputs of Tukey's HSD post-hoc test

	Mean Difference	SEM	q	p-value	Lower 95% CI	Upper 95% CI
Sub. Res.	1.41E-01	2.12E-01	9.41E-01	9.99E-01	-5.22E-01	8.03E-01
Resid. Res.	-2.02E-01	2.12E-01	1.35E+00	9.89E-01	-8.64E-01	4.60E-01
Resid. Sub.	-3.43E-01	2.12E-01	2.29E+00	7.93E-01	-1.01E+00	3.20E-01
Class. Res.	-3.70E-03	2.12E-01	2.47E-02	1.00E+00	-6.66E-01	6.59E-01
Class. Sub.	-1.45E-01	2.12E-01	9.66E-01	9.99E-01	-8.07E-01	5.18E-01
Class. Resid.	1.98E-01	2.12E-01	1.32E+00	9.91E-01	-4.64E-01	8.61E-01
Stair. Res.	-1.33E-01	2.12E-01	8.90E-01	9.99E-01	-7.96E-01	5.29E-01
Stair. Sub.	-2.74E-01	2.12E-01	1.83E+00	9.32E-01	-9.36E-01	3.88E-01
Stair. Resid.	6.87E-02	2.12E-01	4.59E-01	1.00E+00	-5.94E-01	7.31E-01
Stair. Class.	-1.29E-01	2.12E-01	8.65E-01	1.00E+00	-7.92E-01	5.33E-01
Lab. Res.	1.77E-01	2.12E-01	1.19E+00	9.96E-01	-4.85E-01	8.40E-01
Lab. Sub.	3.66E-02	2.12E-01	2.44E-01	1.00E+00	-6.26E-01	6.99E-01
Lab. Resid.	3.79E-01	2.12E-01	2.53E+00	6.87E-01	-2.83E-01	1.04E+00
Lab. Class.	1.81E-01	2.12E-01	1.21E+00	9.95E-01	-4.81E-01	8.43E-01
Lab. Stair.	3.11E-01	2.12E-01	2.08E+00	8.69E-01	-3.52E-01	9.73E-01
Dorm. Res.	-9.88E-02	2.12E-01	6.60E-01	1.00E+00	-7.61E-01	5.64E-01
Dorm. Sub.	-2.40E-01	2.12E-01	1.60E+00	9.69E-01	-9.02E-01	4.23E-01
Dorm. Resid.	1.03E-01	2.12E-01	6.89E-01	1.00E+00	-5.59E-01	7.65E-01
Dorm. Class.	-9.51E-02	2.12E-01	6.36E-01	1.00E+00	-7.57E-01	5.67E-01
Dorm. Stair.	3.44E-02	2.12E-01	2.30E-01	1.00E+00	-6.28E-01	6.97E-01
Dorm. Lab.	-2.76E-01	2.12E-01	1.85E+00	9.29E-01	-9.38E-01	3.86E-01

Lib. Res.	-9.73E-02	2.12E-01	6.50E-01	1.00E+00	-7.60E-01	5.65E-01
Lib. Sub.	-2.38E-01	2.12E-01	1.59E+00	9.70E-01	-9.00E-01	4.24E-01
Lib. Resid.	1.05E-01	2.12E-01	6.99E-01	1.00E+00	-5.58E-01	7.67E-01
Lib. Class.	-9.36E-02	2.12E-01	6.26E-01	1.00E+00	-7.56E-01	5.69E-01
Lib. Stair.	3.59E-02	2.12E-01	2.40E-01	1.00E+00	-6.26E-01	6.98E-01
Lib. Lab.	-2.75E-01	2.12E-01	1.84E+00	9.31E-01	-9.37E-01	3.88E-01
Lib. Dorm.	1.47E-03	2.12E-01	9.81E-03	1.00E+00	-6.61E-01	6.64E-01
Terr. Res.	1.59E-01	2.12E-01	1.07E+00	9.98E-01	-5.03E-01	8.22E-01
Terr. Sub.	1.85E-02	2.12E-01	1.24E-01	1.00E+00	-6.44E-01	6.81E-01
Terr. Resid.	3.61E-01	2.12E-01	2.41E+00	7.42E-01	-3.01E-01	1.02E+00
Terr. Class.	1.63E-01	2.12E-01	1.09E+00	9.98E-01	-4.99E-01	8.25E-01
Terr. Stair.	2.93E-01	2.12E-01	1.96E+00	9.04E-01	-3.70E-01	9.55E-01
Terr. Lab.	-1.80E-02	2.12E-01	1.20E-01	1.00E+00	-6.80E-01	6.44E-01
Terr. Dorm.	2.58E-01	2.12E-01	1.73E+00	9.51E-01	-4.04E-01	9.20E-01
Terr. Lib.	2.57E-01	2.12E-01	1.72E+00	9.53E-01	-4.06E-01	9.19E-01

Table S9: Q Values of All Convergent PMF Model Runs

Run	Q(Robust)	Q(True)	Converged	Steps	Q(true)/Qexp
1	1539.85	4104.15	Yes	516	51.3019
2	1654.74	4655.07	Yes	637	58.1884
3	1837.58	4995.74	Yes	463	62.4468
4	1588.38	3682.20	Yes	511	46.0275
5	1517.73	4120.77	Yes	508	51.5096

6	1588.17	3684.17	Yes	492	46.0521

Table S10: Mapping Rates of Bootstrap Factors to Base PMF Source Factors

	**Factor 1**	**Factor 2**	**Factor 3**	**Factor 4**	**Unmapped**
Boot Factor 1	92	7	0	0	1
Boot Factor 2	7	77	2	9	5
Boot Factor 3	0	0	100	0	0
Boot Factor 4	2	19	7	67	5

Table S11: Concentrations of Target Compounds across Indoor Functional Environments (mg·kg^{-1})

	Restaurant	Subway	Residence	Classroom	Stairway	Terrace	Laboratory	Dormitory	Library
TEHP	3.36	25.88	0.87	14.38	3.47	9.04	17.21	5.87	3.97
EHDPP	5.93	3.99	1.30	6.51	7.53	8.54	7.43	3.47	7.73
TCIPP	1.46	2.20	0.22	2.64	1.07	0.57	8.86	1.38	0.85
TCEP	1.31	3.26	2.76	1.77	1.06	0.41	3.53	1.16	0.90
TPhP	0.09	1.49	0.01	2.29	0.88	7.16	0.45	0.40	0.12
BBOEHEP	0.14	0.50	0.57	0.46	1.30	0.32	0.51	0.65	1.08
AO168	0.21	0.08	0.19	2.20	0.18	0.22	0.60	0.05	0.22
TBOEP	0.03	2.73	N.D.	0.14	0.07	0.13	0.14	0.10	0.06
AO168=O	0.33	0.23	0.32	0.34	0.16	0.36	0.37	0.18	0.36
DBzP	0.25	0.10	0.47	0.19	0.24	0.09	0.08	0.23	0.26
DMTP.Na	0.21	0.17	0.17	0.17	0.29	0.30	0.16	0.23	0.30
DMP	0.16	0.73	N.D.	0.08	0.03	0.07	0.07	0.52	0.07
TDCIPP	0.06	0.57	N.D.	0.33	0.07	0.20	0.39	0.12	0.08
TEP	0.11	0.06	1.04	0.06	0.02	0.10	0.12	0.08	0.01
4-OH-BDE-47	0.16	0.20	N.D.	N.D.	0.05	0.35	0.23	0.17	0.35
TMPP	0.27	0.22	N.D.	0.11	0.01	0.08	0.37	0.01	0.03
TiBP	0.06	0.05	N.D.	0.09	0.08	0.30	0.28	0.06	0.15
TnBP	0.05	0.05	N.D.	0.09	0.07	0.30	0.28	0.06	0.14
AO626	N.D.	N.D.	0.37	0.01	N.D.	N.D.	N.D.	N.D.	N.D.
MDPP	0.02	0.03	0.04	0.06	0.04	0.04	0.02	0.04	0.04

TiPP	0.02	0.04	0.02	0.02	0.02	0.12	0.05	0.03	0.01
TnPP	N.D.	N.D.	0.01	0.01	0.01	0.35	0.14	N.D.	0.01
V6	N.D.	0.01	0.01	0.01	0.01	0.11	0.09	0.01	0.01
TnPPi	N.D.	N.D.	0.06	0.02	0.02	0.01	0.01	0.01	0.01
TDBPP	N.D.	0.01	N.D.	N.D.	N.D.	0.06	0.07	0.01	N.D.
TMP	N.D.	N.D.	0.02	N.D.	N.D.	N.D.	0.01	N.D.	N.D.
3-OH-TnBP	N.D.	N.D.	N.D.	N.D.	N.D.	N.D.	N.D.	N.D.	N.D.
TPHPi	N.D.	N.D.	N.D.	N.D.	N.D.	N.D.	N.D.	N.D.	N.D.

*Notes: N.D. denotes compounds that were not detected. Values displayed as 0.00 indicate quantified concentrations above the limit of quantification (LOQ) that were rounded to zero for tabular presentation and should not be interpreted as non-detects.

Table S12: Summary of Mean, SD, SE and 95% CI for log_{10}-Transformed Total OPE ADD via Ingestion, Inhalation and Dermal Pathways across Four Age Subgroups

		**Mean**	**SD**	**SE**	**Lower 95% CI**	**Upper 95% CI**
ADD_{oral}	Children	8.87E-01	9.69E-03	6.85E-03	9.00E-01	8.73E-01
	Adolescents	5.04E-01	3.61E-02	2.55E-02	5.54E-01	4.54E-01
	Adults	-5.09E-02	6.39E-02	4.52E-02	3.77E-02	-1.39E-01
	Elderly adults	-3.23E-02	3.97E-02	2.81E-02	2.28E-02	-8.74E-02
ADD_{inh}	Children	-3.07E+00	9.69E-03	6.85E-03	-3.05E+00	-3.08E+00
	Adolescents	-3.45E+00	3.61E-02	2.55E-02	-3.40E+00	-3.50E+00
	Adults	-3.18E+00	6.40E-02	4.53E-02	-3.10E+00	-3.27E+00
	Elderly adults	-3.17E+00	3.97E-02	2.81E-02	-3.11E+00	-3.22E+00
ADD_{der}	Children	-1.06E+00	9.69E-03	6.85E-03	-1.05E+00	-1.08E+00
	Adolescents	-1.45E+00	3.61E-02	2.55E-02	-1.40E+00	-1.50E+00
	Adults	-2.06E+00	6.40E-02	4.53E-02	-1.97E+00	-2.15E+00
	Elderly adults	-2.04E+00	3.97E-02	2.81E-02	-1.99E+00	-2.10E+00

Table S13: Results of One-Way ANOVA and Levene's Homogeneity of Variance Test for log_{10}-Transformed Total OPE ADD of Three Exposure Pathways among Four Age Subgroups

			**df**	**SS**	**MS**	**F**	**p-value**
ADD_{oral}	ANOVA test	Between groups	3	1.23E+00	4.11E-01	2.33E+02	6.07E-05
		Within groups	4	7.06E-03	1.76E-03		
	Levene test	Between groups	3	1.48E-03	4.93E-04	2.56E+29	5.10E-59

		Within groups	4	7.71E-33	1.93E-33		
ADD_{inh}	ANOVA test	Between groups	3	1.60E-01	5.33E-02	3.02E+01	3.31E-03
		Within groups	4	7.07E-03	1.77E-03		
	Levene test	Between groups	3	1.48E-03	4.94E-04	1.00E+28	3.32E-56
		Within groups	4	1.97E-31	4.93E-32		
ADD_{der}	ANOVA test	Between groups	3	1.41E+00	4.70E-01	2.66E+02	4.65E-05
		Within groups	4	7.06E-03	1.77E-03		
	Levene test	Between groups	3	1.48E-03	4.94E-04	8.91E+27	4.20E-56
		Within groups	4	2.22E-31	5.55E-32		

Table S14: Full Pairwise Comparison Outputs of Tukey's HSD Post-Hoc Test for Age-Stratified log_{10}-Transformed Total OPE ADD via Three Exposure Routes

		**Mean Difference**	**SEM**	**q**	**p-value**	**Lower 95% CI**	**Upper 95% CI**
ADD_{oral}	Adol. Todd.	-3.83E-01	4.20E-02	1.29E+01	2.81E-03	-5.54E-01	-2.12E-01
	Adu. Todd.	-9.37E-01	4.20E-02	3.16E+01	8.42E-05	-1.11E+00	-7.66E-01
	Adu. Adol.	-5.55E-01	4.20E-02	1.87E+01	6.67E-04	-7.26E-01	-3.84E-01
	Eld. Todd.	-9.19E-01	4.20E-02	3.09E+01	9.10E-05	-1.09E+00	-7.48E-01
	Eld. Adol.	-5.36E-01	4.20E-02	1.81E+01	7.62E-04	-7.07E-01	-3.65E-01
	Eld. Adu.	1.86E-02	4.20E-02	6.26E-01	9.68E-01	-1.52E-01	1.90E-01
ADD_{inh}	Adol. Todd.	-3.83E-01	4.20E-02	1.29E+01	2.82E-03	-5.54E-01	-2.11E-01
	Adu. Todd.	-1.19E-01	4.20E-02	4.01E+00	1.44E-01	-2.90E-01	5.18E-02
	Adu. Adol.	2.63E-01	4.20E-02	8.86E+00	1.14E-02	9.21E-02	4.34E-01

	Eld. Todd.	-1.01E-01	4.20E-02	3.39E+00	2.20E-01	-2.72E-01	7.05E-02
	Eld. Adol.	2.82E-01	4.20E-02	9.48E+00	8.85E-03	1.11E-01	4.53E-01
	Eld. Adul.	1.87E-02	4.20E-02	6.27E-01	9.67E-01	-1.52E-01	1.90E-01
ADD_{der}	Adol. Todd.	-3.83E-01	4.20E-02	1.29E+01	2.81E-03	-5.54E-01	-2.11E-01
	Adu. Todd.	-9.95E-01	4.20E-02	3.35E+01	6.64E-05	-1.17E+00	-8.24E-01
	Adu. Adol.	-6.13E-01	4.20E-02	2.06E+01	4.52E-04	-7.84E-01	-4.42E-01
	Eld. Todd.	-9.77E-01	4.20E-02	3.29E+01	7.16E-05	-1.15E+00	-8.06E-01
	Eld. Adol.	-5.94E-01	4.20E-02	2.00E+01	5.10E-04	-7.65E-01	-4.23E-01
	Eld. Adu.	1.86E-02	4.20E-02	6.26E-01	9.68E-01	-1.52E-01	1.90E-01

Figure S1: Boxplots of Log_{10}-Transformed Mixed OPE Concentrations and Scaled Residual Histogram of PMF Model

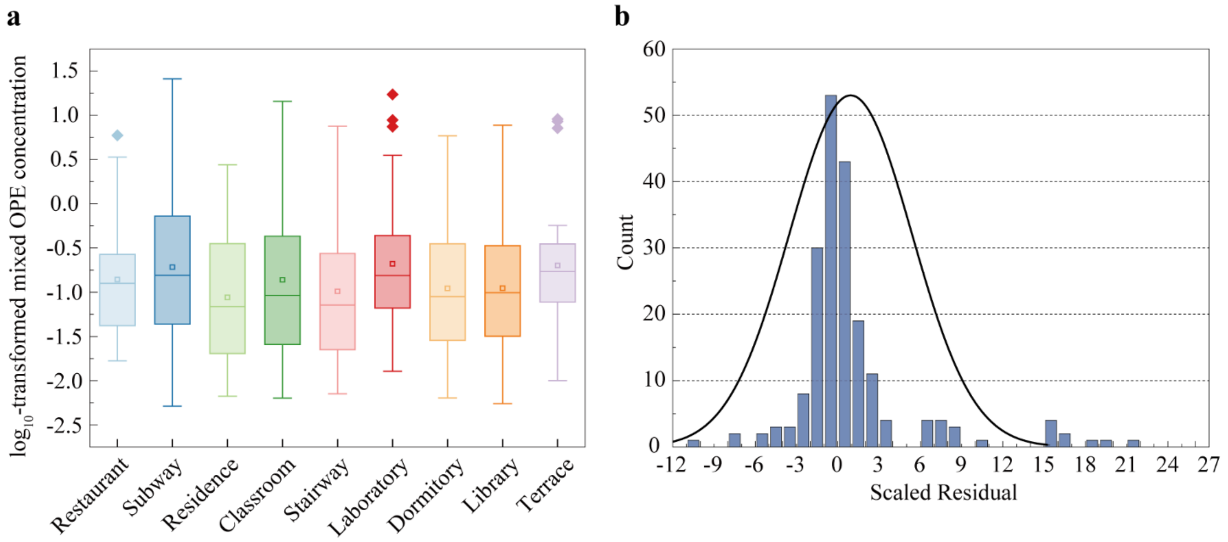

Figure S1. Panel (a) presents boxplots of log_{10}-transformed mixed OPE concentrations in dust samples from nine indoor microenvironments, where error bars denote two-sided 95% confidence intervals. Panel (b) shows the histogram of scaled residuals derived from the PMF

receptor model, with a fitted normal distribution curve overlaid to evaluate the overall fitting performance of the model.
